# Medicinal plants used to treat livestock ailments in Ensaro District, North Shewa Zone, Amhara Regional State, Ethiopia

**DOI:** 10.1186/s12917-022-03320-6

**Published:** 2022-06-22

**Authors:** Asaye Asfaw, Ermias Lulekal, Tamrat Bekele, Asfaw Debella, Eyob Debebe, Bihonegn Sisay

**Affiliations:** 1 College of Agriculture, Natural Resource Management Department, Debre Brehan University, Debre Brehan, Ethiopia; 2 College of Computational and Natural Science, Addis Ababa University, Addis Ababa, Ethiopia; 3 Traditional and Modern Medicine Research Directorate, Traditional Medicine, Ethiopian Public Health Institute, Addis Ababa, Ethiopia

**Keywords:** Ethnoveterinary medicine, Indigenous knowledge, Livestock diseases, Traditional medicine practitioners

## Abstract

**Background:**

In Ethiopia, the majority of animal owners throughout the country depend on traditional healthcare practices to manage their animals' health problems. This ethnoveterinary study was carried out in Ensaro District, North Showa Zone, and Amhara Region, Ethiopia, to identify medicinal plant species used by the local community to treat various livestock ailments.

**Methods:**

To collect ethnobotanical information, a total of 389 informants (283 men and 106 women) were selected. Among these 95 traditional medicine practitioners were purposely chosen, while the remaining 294 were selected through a systematic random sampling method. Ethnobotanical data were collected through semi-structured interviews, participant observation, guided filed walks and focus group discussions. The Informant Consensus Factor (ICF) and Fidelity Level (FL) values, preference, and direct matrix exercise ranking were determined using quantitative methods. Statistical tests were used to compare indigenous knowledge of medicinal plants among different informant groups.

**Results:**

A total of 44 ethnoveterinary medicinal plant species were collected and identified that were distributed across 43 genera and 28 families. The family Solanaceae stood first by contributing 4 species followed by Amaranthaceae, Asteraceae, Euphorbiaceae, Fabaceae and lamiaceae with 3 species each. Thirty- seven (88.09%) medicinal plants were collected from wild habitats, 6 medicinal plant species were collected from home garden. The most frequently used life form was shrubs (23 species, 54.76%) followed by herbs (13 species, 30.95%). The widely used parts of medicinal plants were leaves followed by roots. Prepared remedies were administered through drenching, dropping, smearing, eating, wrapping, fumigating and washing. There was significant difference in the indigenous knowledge of medicinal plants between gender, urban and rural people, general and key informants and among age groups.

**Conclusion:**

Ensaro District is a relatively rich in diversity of ethnoveterinary medicinal plants together with a rich indigenous knowledge in the local communities to collect and effectively utilize for the management different livestock diseases. On the other hand, these days, agricultural expansion, fuel wood collection, cutting plants for fence, furniture and charcoal production are the major threatening factors of these plant resources. Thus, people of the study area must implement in situ and *ex-situ* conservation strategies to ensure sustainable utilization of these species.

**Supplementary Information:**

The online version contains supplementary material available at 10.1186/s12917-022-03320-6.

## Background 

Because of the limited distribution of modern veterinary health care services along with the unaffordable cost of modern drugs which sometimes fail to exert desired benefits, many farmers in developing countries have depended on traditional medicines for the management of diseases of livestock for many centuries [1]. Ethnoveterinary medicine refers to peoples’ beliefs, knowledge, skills, methods, and practices related to animal health that are used extensively in rural regions of developing countries as a primary source of medicine to treat livestock diseases [2, 3].

Ethiopia has one of the largest livestock populations in Africa, which is a major contributor to the overall economy of the country [3]. Thus, livestock production is a crucial element of agriculture in the country [4]. Despite its significant economic benefits, livestock productivity is low [5]. The low output has been attributed in part to the poor health of its animals [6, 7]. Due to the enormous potential of medicinal plants in Ethiopia, traditional herbal medicine is an integral part of local culture and is widely used to treat human and livestock diseases [8, 9].

Although plant-based traditional medicines meet the primary healthcare needs, ethnoveterinary practice is harmed by the acculturation and depletion of plant habitats because of environmental degradation, deforestation, and overexploitation of medicinal plants themselves [10, 11]. Furthermore, ethnoveterinary knowledge and practices have been passed down through generations by oral stories instead of in recorded forms [12]. On the other hand, ethnoveterinary traditions are eroding without adequate documentation and analysis of effective medicinal plants along with the associated indigenous knowledge [1]. Yet, there has been very little effort to assess and document ethnoveterinary medicinal plants in the Amhara Region and Ethiopia. As far as our literature search, there are no ethnoveterinary medicinal plant studies in Ensaro district. Thus, the present study was designed to assess and document ethnoveterinary medicinal plants and associated indigenous practices in Ensaro District, Amhara Region, Ethiopia.

## Methods 

### Description of the study area

**Fig. 1:**
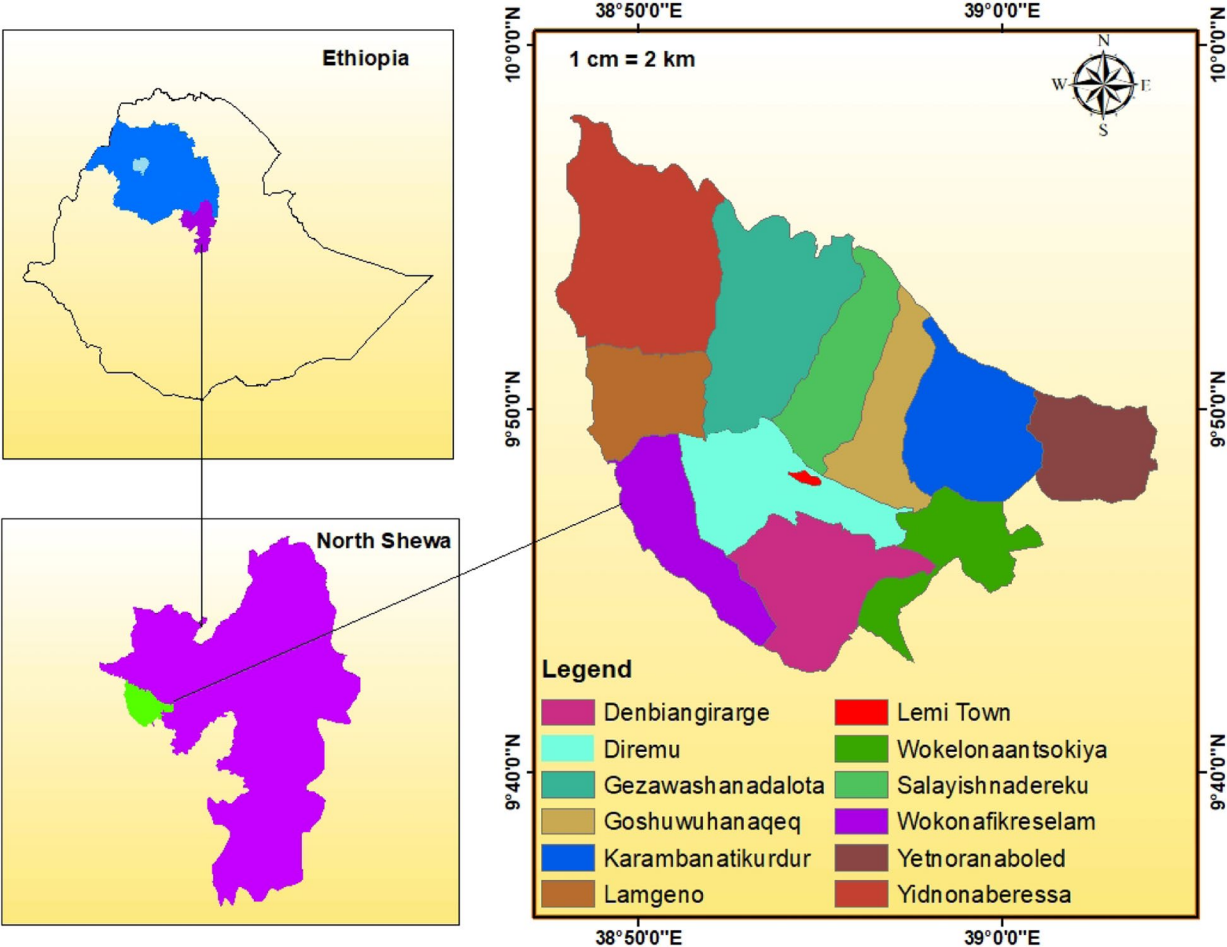
Map of Ethiopia showing Amhara Region and the study district

Ensaro district is geographically located between 9º 35ˈ- 9º 55ˈN and 38º 50’—39º 5’E North Shewa Zone, Amhara Regional State, Ethiopia. The total area of the district is about 44,217.6 ha (Fig. 1). Most of the district is lowland (Fig. 1) with a mean annual rainfall of 1174 mm and a temperature of 17.7OC. The total population is about 74,312 (CSA, 2013), out of which 94.7% inhabit rural areas, deriving their livelihood from mixed agriculture [13]. Ensaro district was selected purposively for the current study. This district is one of the drought-affected districts in the North Shewa Zone of Amhara Regional State. As reported by [13], in the district there is a trend of increasing mean annual temperature and decreasing mean annual rainfall for the three decades. This clearly harms the vegetation of the area and that initiated this research to check the status of cultural knowledge regarding medicinal plants and the plant taxa in the district. There are 14,560 households in the district and the major economic activity of the population is a mixed farming system. The main economic activities of the residents in the study site include crop farming and livestock keeping that together form about 90% of the economic activities followed by cash-earning activities such as merchandising and others [13]. The major farming animals in the district and their estimated numbers of heads of cattle (26,325), goats (12,570), sheep (9,784), donkeys (9410), horses (1206), and chickens (35,901) (N. Mamuye, personal communication, November 22, 2020). Based on the information gathered during the reconnaissance survey, the district has thirteen smaller administrative units at different distances from the administrative center (Lemi Town). All these units were included in this ethnobotanical investigation.

### Study sites and informant selection techniques

Participants were selected based on information collected from Ensaro District Administration Office, health center administrators, agriculture office, and other people in the study area during the reconnaissance survey before the actual data collection. As a result, since the district is a small one, containing only 13 kebeles with 14,560 households, it was necessary to consider all the kebeles (the smallest administrative units) in the district as they are for data collection. In short, all the thirteen kebeles of Ensaro District were included in this investigation.

General informants and knowledgeable traditional medicine practitioners of the district were selected using systematic random and purposive sampling approaches, respectively, in the manner described by [14]. Accordingly, the total number of informants involved in the ethnoveterinary medicinal plant survey was 389 (283 men and 106 women). Informants ranged in age from 20 to 90. Among them, the first 111 were between 20 and 35, 207 were between 36 and 60 and the remaining 71 were 60 and above.

The researchers used peer recommendations from community members, elderly people and knowledgeable inhabitants to select 95 traditional medicine practitioners (77 men and 18 women), While 294 general informants were selected from total households using systematic random sampling method. For this, the list of households was obtained from Ensaro District Agriculture Office in the study area. A total number of 14,560 households was divided by sample size (389) with a result of 37 so that every 37th number was selected from the list to get sampled households.

### Data collection

Ethnobotanical data was collected through semi-structured interview, field observation, guided field walk and focus group discussions [14, 15]. The semi-structured interviews were prepared ahead of time in the English language and translated to Amharic between the interview that is the mother tongue of informants. An informant interview was conducted individually [14] to obtain information about medicinal plant species, parts used, preparation techniques, and common livestock diseases treated, administration routes and dosage. Ethnobotanical data regarding habitat, abundance and the threat of medicinal plants were also collected. The voucher specimens of all the mentioned medicinal plants during the interview were collected from different habitats with the assistance of traditional medicine practitioners. Essential information such as global positioning system data (GPS data), the local name of the plant, habitat, and life forms of plant specimens were recorded. Specimens were numbered, pressed, dried, identified, and deposited at the national herbarium of Ethiopia at Addis Ababa University, Ethiopia. Plant identification was performed using the flora of Ethiopia and Eritrea books [16–18]. The accuracy of identification was confirmed by comparison with the deposited authenticated specimens from Addis Ababa University Herbarium and with the help of supervisors.

### Data analysis

Microsoft excel (2016) and Sigma plot version 14 software were used to analyze ethnobotanical data. The first was used to determine frequency and percentages, while the second was used to carryout statistical analysis, to create graphs and charts. To identify priority species and ensure consistency, ethnobotanical ranking and scoring procedures such as preference ranking, direct matrix ranking exercise, fidelity level and informant consensus factor values were used based on the recommendations of [19, 20].

Fidelity level values were used to determine the relative healing potential of each medicinal plant based on the proportion of informants who agreed on its use against a given disease category [19]. Fidelity level was estimated using a formula: FL (%) = $\frac{{I}_{P}}{{I}_{U}}\times 100,$ Where FL (%) is the fidelity level, IP is the number of respondents who reported the utilization of medicinal plants for a specific main ailment and IU is the total number of respondents who mentioned the same plant for any ailment [20]. Fidelity level is one of the ethnobotanical indices to recommend medicinal plants for their antimicrobial activity test, phytochemical analysis, bioactive chemical isolation and characterization and for drug formulation [21].

The informant consensus factor (ICF) was computed to see the agreement of informants for a plant species in treating a disease using the following formula: $\mathrm{ICF}=$ $\frac{{n}_{ur-{n}_{t}}}{{n}_{ur}-1}$, Where ICF is the informant consensus factor, ${n}_{ur}$ is the number of use citations and ${n}_{t}$ is the number of plant species used following [22].

Direct matrix ranking exercise was used to compare the use diversity of a given plant species using the methods proposed by [14, 15]. The multipurpose uses of medicinal plant species were selected out of the total medicinal plants. Key informants listed the uses of these species. These key informants were asked to assign use values to each species as follows (best = 5, very good = 4, good = 3, less used = 2, least used = 1 and not used = 0). The average values (scores) given to each medicinal plant species were summed up and ranked. Moreover, the ten key informants were also involved in a priority ranking exercise that focused on perceived threatening factors of the medicinal plant species.

In the end, preference ranking from informants' responses on ideas related to disease treatment was analyzed following [14]. Based on their preference ranking, they ranked individually those selected medicinal plants for treating the mentioned ailments following previous scholars [15].

### Ethical consideration

The study was carried out after being approved by the Ethiopian public health institute, traditional medicine directorate. Before conducting semi-structure interview, the participants' consent was obtained and they were assured that their responses would be used only for research purposes, and the information given would be treated with utmost care and confidentiality.

## Results

### Demographic profiles of respondents 

In this study, 389 informants (95 key and 294 general informants) whose age ranged from 20 to 90 years were involved. Regarding educational status, most of them were illiterate (Table 1).

**Table 1:** Demographic characteristics of the informants

Gender	Age groups in years			Educational status		
	**20–35 (young)**	**36–60 (adult)**	**Above 60 (elderly)**	**Modern education**	**Religions education**	**Non-educated**
Men	57	161	65	100	34	149
Women	54	46	6	53	0	53
Total	111(28.5%)	207(53.2%)	71(18.3%)	153(39.33%)	34(8.74%)	202(51.93)

### Traditional knowledge of the community

When the mean number of medicinal plants was compared between men and women using an independent t-test, there was a significant difference (*p* < 0.05). More medicinal plants were listed by men than women. Similarly, there was a statistically significant difference between key and general informants, rural and urban participants (*p* < 0.05). Key informants (traditional medicine practitioners), men and rural residents mentioned a greater number of ethnoveterinary medicinal plants (Table 2). Statistically significant differences among age categories were also be observed (*p* < 0.05) (Table 3). Elderly and adult men mostly possess medicinal plant use knowledge.

**Table 2:** Statistical test of significance and independent t-test on the number of medicinal plants mentioned by informant groups in the Ensaro District

Parameters	Informant groups	N	mean	T-value	*P*-value
Informant types	General informants	294	4.1	2.126	0.035*
	Traditional medicine practitioners	95	4.6		
Gender	Men	283	4.7	9.134	0.0001*
	Women	106	3.1		
Place of residence	Rural	368	3.8	15.76	0.0001*
	Urban	21	1.2		

^*^Significant difference (*P* < 0.05), **t (0.05) (two tailed), df = 388, N = number of informants

**Table 3:** Statistical test of significance using one-way ANOVA test on the number of medicinal plants mentioned by informants' age groups in the Ensaro District

Age groups	The total number of medicinal plants mentioned	Mean ± SD	F-test	*P*-value
Young (19–35 years)	284	2.56 ± 0.85^a^	159.2	0.0001*
Adult (36–60 years)	927	4.48 ± 2.08^b^		
Elderly (above 60 years)	438	6.17 ± 2.77^c^		

^*^Significant difference (*P* < 0.05), **t (0.05) (two tailed), df = 388, N = number of respondents

### Medicinal plants of the study area

The present study documented 44 ethnoveterinary medicinal plants distributed in 43 genera and 28 families, which have been used to treat 16 livestock ailments (Table 4). Nine medicinal plant families were represented by two or more species, while nineteen families were represented by a single species each (Table 5). Solanaceae was the dominant family contributing four species, Amaranthaceae, Asteraceae, Euphorbiaceae, Fabaceae and Lamiaceae each with three species came next. This implies that about 56.84% of families were represented by more than one species.

**Table 4:** List of medicinal plants used for livestock ailments: Family name, scientific name, local name, Life forms, parts used, preparation methods, methods of application, diseases treated, and voucher number

Family	Scientific name	Local name	Life form	Parts used	Preparation methods	Application methods	Diseases treated	Voucher no.00xxAA
Acanthaceae	*Justicia schimperiana* (Hochst. ex Nees) T. Anderson	Sensel	Shrub	Leaf	Crushing and squeezing the juice and mixing with water	Drenching	Jaundice	78
					Crushing and boiling with water	Washing affected part	Rurt (Tail Amputation)	
					Crushing and mixing with water	Drenching	Blackleg	
					Crushing and mixing with water	Drenching	Anthrax	
Alliaceae	*Allium sativum* L	Nech shinkurt	Herb	Bulb	Crushing and mixing with water	Drenching	Blackleg	not collected
				Whole part	Crushing and mixing with water	Drenching	Anthrax	77
Amaranthaceae	*Achyranthes aspera* L	Telenj	Herb	Leaf	Crushing fresh leaves and mixing with water	Adding through both noses	Leech	
					Crushing fresh leaves	smearing	Wound	
	*Cyathula cylindrica* Moq	Arefrafo	Herb	root	Unprocessed root	Wrapping on the tail	Footrot	197
	*Chenopodium ambrosioides* L	Sinign	Herb	Leaf	Crushing with *Solanum villosum* and mixing with water	Dropping to eyes	Eye disease	217
Apiaceae	*Heteromorpha arborescens* (Spreng.) Cham. & Schltdl	Yejib mirkuz	Shrub	Stem bark	Crushing fresh stem bark and squeezing the juice and mixing with water	Drenching through the left nose	Leech	151
Apocynaceae	*Carissa spinarum* L	Agam	Shrub	root	Crushing fresh roots and mixing with water	Drenching through mouth	Lung disease	201
Asclepiadaceae	*Calotropis procera* (Aiton) Dryand	Qebo	Shrub	Stem juice	Crushing and squeezing the juice	smearing	Tumour	93
Asteraceae	*Inula confertiflora* A. Rich	Gobez teqes	Shrub	Leaf	Crushing fresh leaves, filtering and mixing with water	Dropping to eyes	Eye disease	63
	*Solanecio gigas* (Vatke) C. Jeffrey	Yeshikoko gomen	Shrub	Leaf	Crushing fresh leaves and mixing with water	Drenching through mouth	‘mitch” (febrile illness)	112
	*Vernonia amygdalina* Delile	Grawa	Shrub	Leaf	Crushing fresh leaves and mixing with water	Drenching through mouth	Jaundice	103
Balsaminaceae	*Impatiens tinctoria* A.Rich	Yebereha shimbra	Herb	root	Crushing fresh roots and mixing with water	Drenching through mouth	Blackleg	220
Berberidaceae	*Berberis holstii* Engl	Yeset af	Shrub	Leaf	Crushing fresh leaves and mixing with water	Dropping to eyes	Eye disease	150
Buddlejaceae	*Buddleja polystachya* Fresen	Afar	Shrub	Leaf	Crushing fresh leaves and mixing with water	Drenching through noses	Leech	199
Caryophyllaceae	*Silene macrosolen* Steud. ex A. Rich	Wogert	Herb	root	Powdering dried roots	Fumigating the room	Blackleg	155
Celastraceae	*Maytenus senegalensis* (Lam.) Exell	Atat	Shrub	Leaf	Crushing fresh leaves and filtering using cotton cloth	adding three drops of filtrate to eyes	Eye disease	21
Cucurbitaceae	*Cucumis ficifolius* A. Rich	Yemdir embuay	Climber	root	Crushing and mixing with water	Drenching through mouth	Blackleg	11
				Fruit	Crushing ripened fruits	Smearing	Rurt (Tail Amputation)	
				root	Cutting inot small pieces using horn knife	Wrapping on the tail	Footrot	
					Powdering dried roots and baking with *Lepidium sativum*	Eating	Rabies	
Cupressaceae	*Juniperus procera* Hochst. ex Endl	Yabesha tid	Tree	Leaf	Crushing fresh leaves and mixing with water	Drenching through mouth	Blackleg	88
Euphorbiaceae	*Croton macrostachyus* Hochst. ex Del	Bisana	Tree	Leaf	Crushing fresh leaves and mixing with water	Drenching through mouth	Footrot	25
	*Euphorbia abyssinica* J.F.Gmel	Qulqual baledemu	Shrub	Latex	Extracting the juice and mixing with milk	Drenching through mouth	Rabies	167
	*Ricinus communis* L	Gulo	Herb	leaf	Crushing fresh leaves and mixing with water	Dropping to eyes	Eye disease	186
Fabaceae	*Acacia etbaica* Schweinf	Derie	Tree	leaf	Crushing fresh leaves and mixing with water	smearing	Tumour	47
	*Calpurnia aurea* (Ait.) Benth	Digita	Shrub	Leaf	Crushing fresh leaves	smearing	Ticks	18
	*Millettia ferruginea* (Hochst.) Bak	Birbira	Tree	Stem bark	Crushing fresh stem bark and squeezing the juice and mixing with water	Drenching through mouth	Tumour	193
Lamiaceae	*Leonotis ocymifolia* (Burm.f.) Iwarsson	Ras kebdo	Shrub	root	Crushing fresh roots and mixing with water	Drenching through mouth	Anthrax	79
	*Salvia nilotica* Juss. ex Jacq	Hulgeb	Herb	root	Crushing fresh roots and mixing with water	Drenching through mouth	Blackleg	31
				Whole part	Crushing the whole part and mixing with water	Drenching through mouth	Footrot	
	*Premna schimperi* Engl	Checho	Shrub	Leaf	Crushing fresh or powdering dried leaves and mixing with water	Dropping	Eye disease	23
Myrtaceae	*Eucalyptus globulus* Labill	Nech bahir zaf	Tree	Leaf	Crushing fresh leaves, adding salt and mixing with water	Drenching through mouth	Blackleg	171
Olacaceae	*Jasminum abyssinicum* Hochst. ex DC	Tembelel	Shrub	Leaf	Crushing fresh leaves and adding milk	Drenching through mouth	Jaundice	82
Peraceae	*Clutia abyssinica* Jaub. & Spach	Fiyelefej	Shrub	Leaf	Crushing fresh leaves and mixing with water	Drenching through mouth	Diarrhoea	53
Phytolaccaceae	*Phytolacca dodecandra* L' Herit	Mekan endod	Shrub	Root	Powdering dried roots and baking with traditional food (Injera)	Eating	Rabies	8
Poaceae	*Cynodon dactylon* (L.) Pers	Serdo	Herb	Root	Powdering dried root and baking with bread	Eating	Rh-factor	131
	*Pennisetum thunbergii* Kunth	Ssindedo	Herb	Roo	Powdering dried roots and baking with bread	Eating	Rh-factor	160
Polygonaceae	*Rumex nepalensis* Spreng	Lut	Herb	root/leaf	Crushing fresh material and mixing with water	Drenching through the mouth	Blackleg	127
Rosaceae	*Rubus steudneri* Schweinf	Enjori	Shrub	Leaf	Crushing fresh leaves and mixing with water	Drenching through the mouth	Swelling on any body part	234
Rutaceae	*Citrus aurantiifolia* (Christm.) Swingle	Yabesha lomi	Shrub	Fruit	Crushing and squeezing the juice	Drenching through both noses	Leech	154
	*Ruta chalepensis* L	Tenadam	Herb	Leaves/fruits	Crushing fresh plant material and mixing with water	Drenching through the mouth	Blackleg	not collected
Scrophulariaceae	*Verbascum sinaiticum* Benth	Yahya joro	Herb	Root	Crushing fresh roots and mixing with water	Drenching through the mouth	Blackleg	153
Solanaceae	*Solanum villosum* Mill	Derekus	Shrub	leaf	Crushing fresh leaves and mixing with water	dropping	Eye disease	2
	*Solanum marginatum* L. f	Geber embuay	Shrub	Fruit	Crushing ripened fruits and taking out the juice	dropping	Eye disease	80
				Fruit	Crushing fresh and ripened fruits	smearing	Rurt (Tail Amputation)	
	*Nicotiana tabacum* L	Tinbaho	Herb	leaf	Crushing fresh leaves and mixing with water	Drenching through the mouth	Blackleg	157
					Crushing fresh leaves and mixing with water	Drenching through left nose	Leech	
	*Capsicum annuum* L	Mitmita	Herb	Fruit	Powdering dried fruits and mixing with water	Drenching through the mouth	Blackleg	not collected
Verbenaceae	*Lippia adoensis* Hochst	Yelam kessie	Shrub	leaf	Crushing fresh leaves, adding with water and filtering by cotton cloth	Dropping	Eye disease	73
	*Clerodendrum myricoides* (Hochst.) R.Br. ex Vatke	Misirch	Shrub	leaf	Crushing fresh leaves and mixing with water	Drenching throuhg mouth	Anthrax	101

**Table 5:** Diversity of medicinal plants in each plant family

No	Plant families	Number of species	percentage
1	Solanaceae	4	9.09%
2	Amaranthaceae	3	6.82%
3	Asteraceae	3	6.82%
4	Euphorbiaceae	3	6.82%
5	Fabaceae	3	6.82%
6	Lamiaceae	3	6.82%
7	Poaceae	2	4.55%
8	Rutaceae	2	4.55%
9	Verbenaceae	2	4.55%
10	The rest(19 families)	19	43.2%
11	Total	44	

### Growth forms of medicinal plants 

**Fig. 2:**
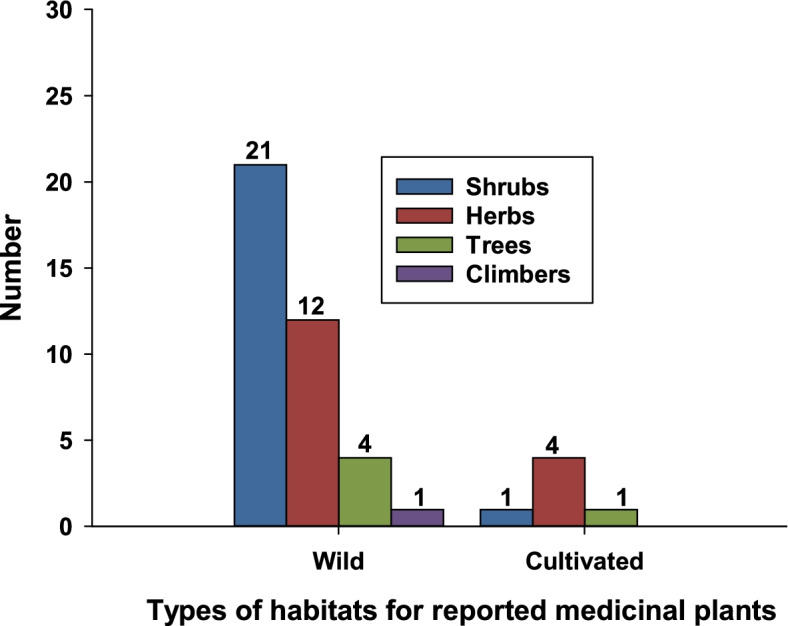
Life forms/habitats of documented ethnoveterinary medicinal plants

The analysis of the growth forms of medicinal plants indicated that shrubs constitute the highest number of species whereas herbs, trees and climbers came after, respectively. About 86.4% of medicinal plants were collected from wild habitats and very few were cultivated in the home garden (Fig. 2).

### Parts of medicinal plants used

**Fig. 3:**
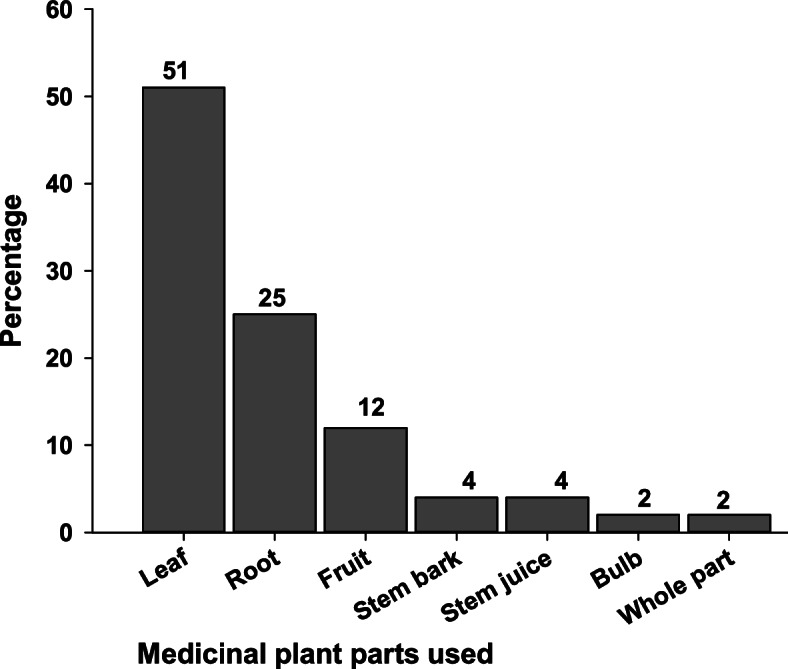
Plant part(s) used for recipe preparation

The study revealed that diverse types of plant parts were used to treat various ailments of livestock either in combination or alone. The analysis of the collected information showed that leaves were the widely used plant parts succeeded by roots, fruit, and so on (Fig. 3).

### Conditions of preparation

**Fig. 4:**
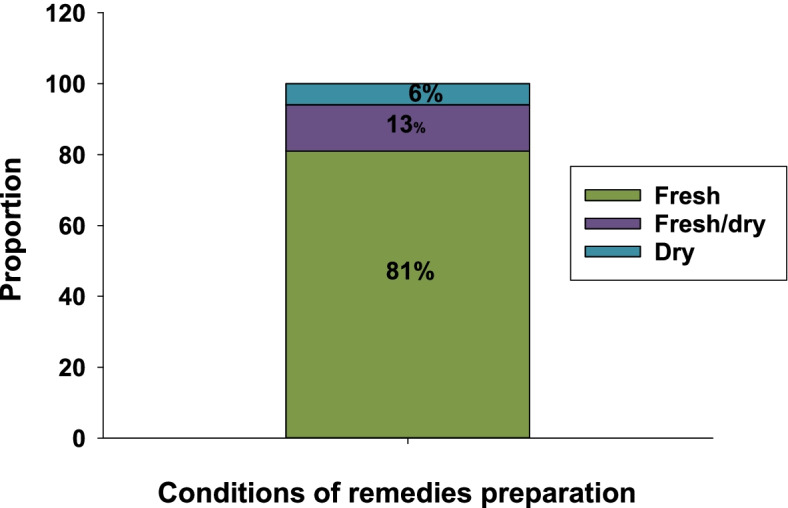
Conditions of traditional herbal medicines preparations

Traditional herbal medicines were prepared in fresh, dry, or both dry and fresh conditions of plant parts. The Marjory of traditional herbal medicines were prepared in fresh form, fresh/dry form and only in dry form in the order given (Fig. 4).

### Methods of application

**Fig. 5:**
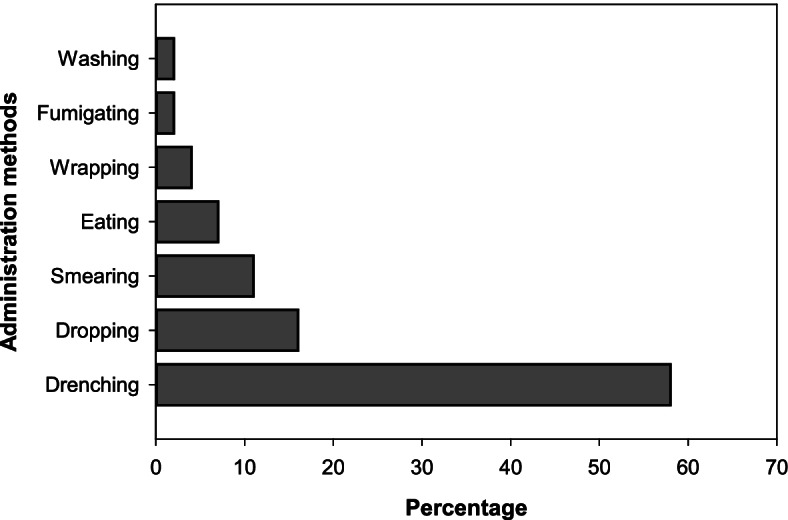
Methods of plant medicine applications in Ensaro District

This study reported a wide range of traditional remedy applications. Adding through the mouth (drenching) was the most common, accounted for the highest proportion, followed by dropping and smearing (Fig. 5).

### Ingredients added during remedy preparation

**Fig. 6:**
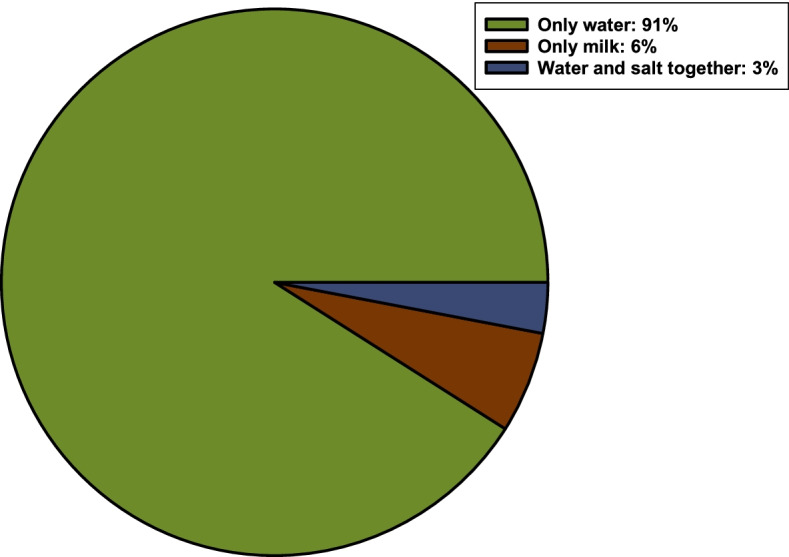
Ingredients added during remedy preparations

Solvents and ingredients are required for the preparation of traditional medicines. Water was the most common solvent to prepare herbal remedies in the home (Fig. 6).

### Routes of applications

**Fig. 7:**
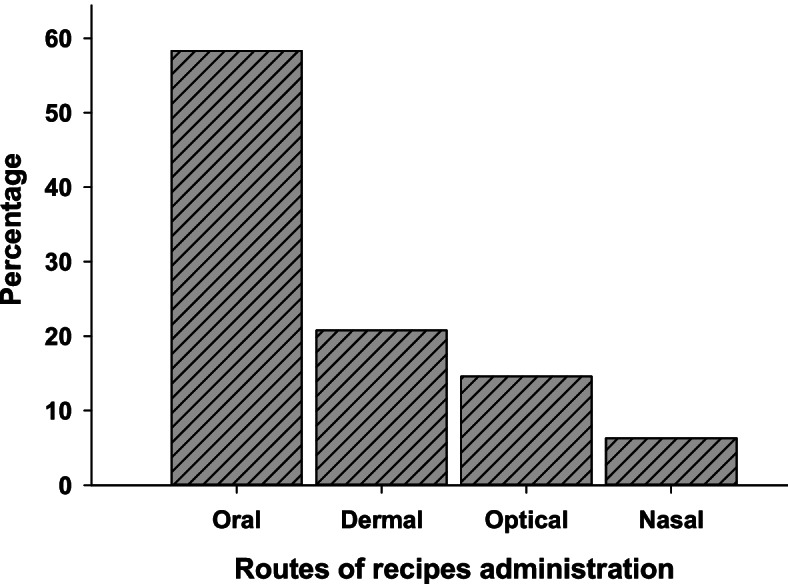
Routes of recipes administration

The current study indicated different routes for the applications of traditional herbal medicines for the treatment of various types of livestock ailments and diseases. Oral administration is the most used route, which is followed by dermal application while the optical and nasal routes contributed least (Fig. 7).

### Ailments of livestock treated by medicinal plants 

In this area, 44 medicinal plants were collected and identified for the treatment of 16 types of livestock health disorders (Table 4). Several medicinal plants were discovered to treat a single livestock ailment. For example, blackleg is treated by Justicia schimperiana (Hochst. ex Nees) T. Anderson, Allium sativum L., Silene macrosolen Steud. ex A. Rich., Cucumis ficifolius A. Rich., Salvia nilotica Juss. ex Jacq., Eucalyptus globulus Labill, Rumex nepalensis Spreng., Ruta chalepensis L., and so on. On the other hand, a single medicinal plant species can treat a number of livestock diseases in the study area. For instance, Justicia schimperiana (Hochst. ex Nees) T. Anderson is used to treat Jaundice, Tail amputation, Blackleg and anthrax (Table 4).

### Preference ranking 

The five most reported medicinal plants for effective treatment of blackleg that were frequently reported in the study area were selected for preference ranking exercise. Ten key informants were asked to rank the given medicinal plants based on their usefulness. They were also asked to give numbers 5 for effective and 1 for less effective medicinal plants. As a result, Cucumis ficifolius A. Rich. was ranked first and Verbascum sinaiticum Benth. ranked second, whereas Ruta chalepensis L. was ranked last (Table 6).

**Table 6:** Simple preference ranking values of five medicinal plants used to treat blackleg

Name of medicinal plants	Informants from (A-J)										Total	Rank
	A	B	C	D	E	F	G	H	I	J		
*Cucumis ficifolius* A. Rich	4	5	4	5	5	4	4	5	5	5	46	1^st^
*Verbascum sinaiticum* Benth	5	3	4	4	3	2	1	4	5	4	35	2^nd^
*Allium sativum* L	5	3	2	4	3	3	4	5	2	3	34	3^rd^
*Salvia nilotica* Juss. ex Jacq	3	5	3	4	2	1	3	2	1	2	26	4^th^
*Ruta chalepensis* L	2	4	2	3	2	1	2	3	3	3	25	5^th^

### The relative healing potential of medicinal plants used to treat livestock diseases 

The relative healing potential of medicinal plants was computed to identify the most preferred medicinal plant species used to treat livestock ailments in the study area. Hence, Cynodon dactylon (L.) Pers., Inula confertiflora A. Rich., Nicotiana tabacum L., Verbascum sinaiticum Benth., Cucumis ficifolius A. Rich., Phytolacca dodecandra L’ Herit., Achyranthes aspera L., Clutia abyssinica Jaub. & Spach, Calpurnia aurea (Ait.) Benth., and Justicia schimperiana (Hochst. ex Nees) T. Anderson had the highest fidelity level values that were used as a sign of their healing potential in the study area (Table 7).

**Table 7:** FL values of the 15 most referenced medicinal plants

Medicinal plant species	Diseases	I_p_	I_u_	Fidelity level values
*Cynodon dactylon* (L.) Pers	Rhesus factor	8	8	100
*Inula confertiflora* A. Rich	Eye disease	68	70	97.1
*Nicotiana tabacum* L	Leech	38	40	95
*Verbascum sinaiticum* Benth	Tumor	29	31	93.5
*Cucumis ficifolius* A. Rich	Foot rot	28	34	82.4
*Phytolacca dodecandra* L' Herit	Rabies	29	36	80.6
*Achyranthes aspera* L	Wound/bleeding	12	15	80
*Clutia abyssinica* Jaub. & Spach	Diarrhea	18	23	78.3
*Calpurnia aurea* (Ait.) Benth	External parasites	28	37	75.7
*Justicia schimperiana* (Hochst. ex Nees) T. Anderson	Jaundice	19	27	70.4
*Solanecio gigas* (Vatke) C. Jeffrey	‘’Chirt” (trypanosomiasis	10	16	62.5
*Clerodendrum myricoides* (Hochst.) R.Br. ex Vatke	Anthrax	41	69	59.4
*Cucumis ficifolius* A. Rich	Tail amputation	16	29	55.2
*Carissa spinarum* L	Lung disease	6	11	54.5
*Salvia nilotica* Juss. ex Jacq	blackleg	31	62	50

### Informant consensus factor 

To compute informant consensus factor values (ICF), diseases of the study area were grouped into eight categories based on [23]. As a result, the digestive system scored the highest ICF value, followed by dermal and parasitic caused diseases, respectively (Table 8). Among the digestive system diseases, blackleg was the top-recorded livestock health disorder in the study district veterinary office. It was traditionally treated with 13 medicinal plant species such as Salvia nilotica Juss. ex Jacq, Cucumis ficifolius A. Rich., Allium sativum L., Verbascum sinaiticum Benth., Ruta chalpensis L., Justicia schimperiana (Hochst. ex Nees) T. Anderson and so son (Table 4).

**Table 8:** ICF values of traditional medicinal plants for treating livestock ailments in Ensaro district

Diseases categories	Ailments	N_ur_	N_t_	ICF values
Digestive system	Blackleg, Anthrax and Diarrhoea	124	18	0.86
Dermal	Wound, swelling, Tumour, Tail Amputation and foot rot	74	12	0.84
Parasitic causes	Leech and ticks	31	6	0.83
Respiratory diseases	Lung disease and “mitch” (fibril illness)	5	2	0.75
Viral causes	Rabies and Jaundice	20	6	0.74
Reproductive disease	Rhesus factor	4	2	0.67
Sense organ diseases	Eye disease	18	9	0.53

### Direct matrix ranking exercise 

In addition to medicinal values, the local people used plants for other different purposes, such as fuelwood, fence, food, charcoal, and furniture. The result of direct matrix ranking revealed that Juniperus procera, Acacia etbaica, Croton macrostachyus, Silene macrosolen, Buddleja polystachya, Eucalyptus globulus, Millettia ferruginea, Carissa spinarum, Citrus aurantiifolia were ranked first to ninth, respectively. Similarly, the seven use-values reported on six selected plant species were summed up and ranked. The result showed that fuelwood collection, fence, farm implements, house construction, medicine, and food were ranked first, second, third, fourth, fifth, sixth, and seventh, respectively (Table 9).

**Table 9:** Direct matrix ranking of six plant species by twelve informants based on seven use criteria (best = 5; very good = 4; good = 3; less used = 2; least used = 1 and no value = 0)

Plant species	Use diversity								
	Medicine	Agriculture land expansion	Fence	Fuelwood	Furniture	Charcoal	Food	Total	Rank
*Juniperus procera* Hochst. ex Endl	3	4	4	5	5	3	3	27	1st
*Millettia ferruginea* (Hochst.) Bak	3	4	5	4	1	3	4	24	2nd
*Croton macrostachyus* Hochst. ex Del	4	5	3	5	5	0	0	22	3rd
*Acacia etbaica* Schweinf	1	5	3	5	5	0	0	19	4th
*Buddleja polystachya* Fresen	3	3	1	5	5	1	0	18	5th
*Carissa spinarum* L	2	3	2	0	0	4	3	14	6th
*Citrus aurantiifolia* (Christm.) Swingle	3	2	4	1	1	1	0	12	7th
*Eucalyptus globulus* Labill	1	4	1	2	3	0	0	11	8th
Total	20	30	23	27	25	12	10		
Rank	5th	1st	4nd	2rd	3th	6th	7th		

## Discussion

In the present study, it was found that the men were more knowledgeable than the women as far as the use of medicinal plants is concerned. This is perhaps due to the fact that in most part Ethiopia, the major responsibilities of women are restricted to the home and homestead areas while most of the outdoor activities are done by men [24], which exposes them to sharing knowledge with others. Furthermore, traditional healers have a longstanding practice in preferring their sons to their daughters in conveying their invaluable indigenous knowledge on the use of medicinal plants [25, 26]. This aligns with the research results of [27] which indicated that more than ninety percent of ethnoveterinary practitioners were men. Moreover, the study carried out by [28] in Enarj Enawga District, East Gojjam Zone, Amhara Region, Ethiopia also revealed consistent findings. This indicated that indigenous knowledge is not equally distributed between genders in the country. In contrast to this, [29] stated that regarding “Local knowledge of medicinal plants in three artisanal fishing communities (Itapoá, Southern Brazil), according to gender, age, and urbanization”, women are more knowledgeable than men. Thus, based on this, it can be deduced that such knowledge differences between genders in different parts of the world could be the result of cultural disparities.

Another finding of the current study is high diversity of ethnoveterinary medicinal plants. The varied agro-ecology and climatic conditions that supported a variety of plant species may be attributed to the high diversity of documented medicinal plant species in Ensaro area. Therefore, the existence of diverse medicinal plant species in Ensaro district is possibly the main source of beneficial indigenous knowledge used in the community. This indicated that regardless of its vegetation cover, the study area has a good status of ethnoveterinary medicinal plants and associated indigenous knowledge. In developing countries, medicinal plants have remained the most economically affordable and easily accessible source of treatment for a variety of livestock health problems [30–33].

The number of medicinal plants documented in this paper was comparable with other similar previous studies in different parts of Ethiopia and other countries. In a study conducted in Ada’ar District, Afar Regional State, Ethiopia, 49 ethnoveterinary medicinal plants were identified, documented, and published by [8]. Similarly, another report by [34] of the study in Dabo Hana District, West Ethiopia also showed that they identified and recorded 48 ethnoveterinary medicinal plants which used to treat 22 types of livestock diseases. In Ankober District, North Shewa Zone, Amhara Regional State, 51 medicinal plants distributed in 50 genera and 35 families were reported by [35]. Furthermore, an ethnoveterinary study conducted by [36] in Kenya exhibited that the Kikuyus people are using 40 medicinal plants to treat different kinds of cattle diseases. These findings demonstrated that ethnoveterinary medicines are still important in the prevention and control of livestock diseases in Ethiopia and other developing countries.

Most of the ethnoveterinary medicinal plants identified and documented in this study were also reported in previous studies conducted in different parts of Ethiopia. Among the 44 medicinal plant species documented by current study, 7 species in Enarj Enawga District, East Gojjam Zone, Amhara Region, Ethiopia by [37], 20 species in Bale Mountains National Park, Ethiopia by [38], 9 species in different selected districts of Southern Ethiopia by [9], 9 species in Seharti-Samre district, Northern Ethiopia by [12], 16 species Ankober District, North Shewa Zone, Amhara Region, Ethiopia by [35], 15 species in Abergelle, Sekota and Lalibela districts of Amhara region, Northern Ethiopia by [39], 8 species in Leka Dullecha District, Western Ethiopia by [40], 8 species in South Wollo Zone, Amhara region, Ethiopia by [41] were documented. These findings revealed that there is the widespread use of ethnoveterinary medicinal plants and associated indigenous knowledge in controlling and preventing livestock diseases in Ethiopia. Additionally, it implies that similar medicinal plant species are used to manage diseases of livestock in different geographical locations. This in turn implies that indigenous knowledge is shared among communities from various geographic places.

The analysis of our data also showed that Solanaceae, Asteraceae, Euphorbiaceae, and Lamiaceae accounted for the largest share of the reported ethnoveterinary medicinal plant families. Similar investigations conducted in Ethiopia [35, 37, 42] and elsewhere [36] documented the dominancy of these families in the traditional medicines to treat livestock diseases. This indicated the wider distribution and abundance of these plant families in east Africa. Furthermore, the widespread use of species from these families could be linked to their more effective treatments against diseases [43].

Many of the documented plants in the Ensaro District were shrubs that could be linked to an abundance of shrubby plant species in the study area. Recent ethnobotanical studies have reported the most abundant use of shrubs in ethnoveterinary medicines [8, 35, 44]. Other researchers [9, 37, 41, 45] found that herbaceous species dominated ethnoveterinary medicine preparations in different regions of Ethiopia. This difference in the use of different life forms of ethnoveterinary plants revealed the existence of different agro-ecology in different parts of Ethiopia.

Similar to other ethnobotanical inventories conducted in different regions of the country [35, 37], the majority of ethnoveterinary medicinal plants in the present study area were collected from wild habitats. In Ethiopia, it is popular to use wild or uncultivated medicinal plants to treat livestock and human diseases [8, 46]. This implies that the domestication of medicinal plants is not yet the tradition of users in the country. This may lead to overexploitation and threaten these plant resources, as there are no conservation actions.

According to our findings, the leaves were the most commonly used plant parts in the study district for the preparation of remedies. In line with this, other studies in Ethiopia found that leaves were the most frequently used plant parts for the preparation of recipes [11, 35, 47]. The main reason why many traditional medicine practitioners used leaf parts for remedial preparation instead of other parts is that they are more accessible and help to prevent extinction of the plant species [48, 49].

To make effective and efficient treatments, the majority of ethnoveterinary practitioners in the study district used fresh plant materials. The use of fresh plant materials for remedy preparations is well documented elsewhere in Ethiopia [9, 35, 41, 50] as fresh plant materials maintain the majority of bioactive compounds when compared to dry plant materials which lost volatile and important secondary metabolites.

The majority of participants confirmed that the bulk of the preparations were made by crushing and mixing with water, which is consistent with prior findings from other studies [9, 51]. Most participants reported that the main means of administering medicine was oral route, which agrees with the findings of [50, 52].

The calculation of fidelity level of medicinal plants also found that Cynodon dactylon (L.) Pers., Inula confertiflora A. Rich., Nicotiana tabacum L., and Verbascum sinaiticum Benth had highest fidelity levels. According to [53], fidelity level is a measure of the healing ability of medicinal plants. Therefore, medicinal plants having a high fidelity level indicated that they are speculated to be effective in their curing potential and can be a good candidate for future further detailed investigations.

In addition, the calculation of informant consensus factor values showed that prevalent diseases in the study area had higher informant consensus factor values and less prevalent diseases showed smaller informant consensus values. A report by [54] indicated that plants showing higher informant consensus values are thought to have more biologically active secondary metabolites as compared to plants with less informant consensus values. The higher informant consensus values also suggested that people in the community share knowledge about the most significant medicinal plant species for the treatment of most common ailments. Whereas, the lower values of informant consensus factors also indicated that the willingness to share indigenous knowledge among traditional healers is minimum. This is probably due to the belief that healing power of medicinal plants is reduced if the secret is revealed to others [55, 56]. Furthermore, traditional healers living in different habitats may use different medicinal plant species to treat the same diseases.

The preference ranking exercise helped in determining which medicinal plant species are most used to treat blackleg that is frequently reported in the study area. As a result, Cucumis ficifolius A. Rich., Verbascum sinaiticum Benth., Allium sativum L., Salvia nilotica Juss. ex Jacq., and Ruta chalepensis L. had the highest scores and were identified as the most effective treatments for this disease. Future research on the bioactive components of these medicinal plant species against blackleg causing germs may also lead to good result.

The results of a direct matrix ranking exercise revealed that the highest values (ranks) for several multipurpose ethnoveterinary medicinal plants in the study area, such as Juniperus procera Hochst. ex Endl., Millettia ferruginea (Hochst.) Bak, Croton macrostachyus Hochst. ex Del., Acacia etbaica Schweinf., Buddleja polystachya Fresen., and Carissa spinarum L. This result suggested that such medicinal plants are overexploited for non-medicinal purposes rather than for their reported medicinal values. Overharvesting multipurpose medicinal plants for agricultural land expansion, fuelwood collection, furniture production, fence, house construction, charcoal production, and other purposes were identified as contributing factors to the depletion of these species in the study area. As a result, the findings require immediate conservation actions alongside awareness creation to protect the rapidly declining multipurpose ethnoveterinary medicinal plant species of the study area. The same results showing the highest exploitation of multipurpose ethnoveterinary medicinal plants have been documented from different regions of Ethiopia [40] and elsewhere [57, 58].

## Conclusion

Ensaro District is relatively rich in the diversity of ethnoveterinary medicinal plant species. Forty-four medicinal plants were collected and identified. These ethnoveterinary medicinal plant species were used by people of Ensaro district to treat 16 types of ailments of livestock. Blackleg, eye disease, footrot, leech, jaundice, rabies, tail ampulation, tumor and anthrax were frequently occurring livestock diseases. This showed that the local people are highly dependent on ethnoveterinary medicinal plants despite the fact that the distribution of modern health services are increasing. In the study area, there were significant knowledge differences between men and women, general and key informants, rural and urban inhabitants and among different age groups of informants. Information on preference ranking, fidelity level and informant consensus values of documented ethnoveterinary medicinal plants would be necessary for future antimicrobial activity and phytochemical studies, while direct matrix ranking exercise values call urgent attention on conservation of multipurpose medicinal plants in the study area.

## Supplementary Information

**Additional file 1. **

**Additional file 2. **

## Data Availability

The authors declare that all other data supporting the findings of the study are available within the article and its supplementary information files.
